# Interpretation inaccuracies in pain communication between Hmong patients and clinicians in the USA: A conversation analysis informed study

**DOI:** 10.1016/j.socscimed.2025.118502

**Published:** 2025-08-20

**Authors:** Binh Ta, Mai Nhia Yang, Alice Hu, Maichou Lor

**Affiliations:** a Monash University, Faculty of Medicine Nursing and Health Sciences, Australia; b University of Wisconsin-Madison, School of Nursing, United States

## Abstract

Effective pain communication between patients and providers is imperative in order for providers to understand how to properly manage pain. Limited English proficiency patients, who have to rely on interpreting services, experience poorer pain management than the general population. This study aims to explore the interactional features of pain communication that account for interpretation inaccuracies in consultations involving Hmong patients.

We adopted a conversation analysis informed approach to analyze audio recordings of 27 triadic consultations involving 27 Hmong patients, four interpreters, and 20 clinicians in primary care clinics in Wisconsin, USA.

We found two features of Hmong patients’ pain communication that accounts for interpretation inaccuracies. First, Hmong patients frequently expressed their pain through storytelling, which cause problems when interpreters summarize and omit parts of the stories. Second, Hmong patients frequently used vocal features (such as elongation, voice intensity, and repetition of key lexicons) to convey pain severity, the level of importance of the pain concern, and associated emotions. However, the meaning of these features was not often interpreted accurately, which may have contributed to clinicians missing opportunities to display empathy and address patients’ concerns.

Problems arise when interpreters omit parts of patients’ pain stories and fail to convey the meaning of vocal features. Training is needed to inform interpreters about how pain stories are contextually constructed, and how vocal features can be used to express various aspects of pain.

## 1. Introduction and background

### 1.1. Introduction

Pain is a subjective experience influenced by social and cultural factors (Berger and Luckmann, 2023), making it complex and challenging to diagnose and manage. Language barriers further hinder effective communication during pain assessment (Lor et al., 2024b). Research show that patients with limited English proficiency (LEP), defined as those who speak English less than well (Migration Policy Institute, n.d.), experienced inadequate communication about pain with their clinicians (Lor et al., 2024a). Specifically, inadequate communication in these situations is often exacerbated by poor medical interpretation during interpreter-mediated consultations between LEP patients and clinicians (Lor et al., 2024a; Kimberlin et al., 2004).

Medical interpreting is typically done by trained professionals in clinical settings (Ji and Laviosa, 2020). Occasionally, bilingual healthcare workers may serve as interpreters, while in some instances, untrained individuals, such as family members or volunteers, are used (Villanueva, 2023). Although professional medical interpreters are the standard of care for patients with LEP, medical interpretation errors are frequently reported in healthcare settings. Observational studies using video and audio recordings of Spanish, Hmong, and Japanese interpretations revealed that omissions and editorializations (where interpreters insert their own interpretations of words or phrases spoken by patients and clinicians) are the most common sources of inaccuracies (Anazawa et al., 2012; Flores et al., 2003; Nápoles et al., 2015). For instance, professional medical interpreters omitted medical information in 35 %–47 % of clinical encounters (Lor and Chewning, 2016). Errors with potential consequences are found to be more common among ad hoc medical interpreters (22 %) compared to professional interpreters (12 %) (Flores et al., 2012). Such inaccuracies can lead to strained clinician-patient relationships (Cox et al., 2019) and suboptimal medical care (Lor et al., 2024).

Research has indicated several factors contributing to inaccurate interpretations, including patient, clinician, and interpreter characteristics, patient characteristics such as age, gender, and communication skills can impact effective patient-interpreter-clinician communication (Malhotra et al., 2017). Similarly, the interpreter’s social factors—including age, race, ethnicity, gender, and socioeconomic status—affect their level of engagement during patient-clinician interactions (Angelelli, 2004). LEP patients have reported that interpreters’ proficiency in English medical terminology and patients’ fluency influences the accuracy of message transmission (Flores et al., 2003; Lor and Chewning, 2016). Additionally, interpreters’ understanding of task relevance (Bolden, 2000), the perception that clinicians prefer concise answers, and a potential lack of experience may play a role (Flores et al., 2012; Kai, 2013). Time constraints during clinic visits may also contribute to the omission of information. Despite these factors, medical interpreters are not trained in pain. Research is needed to better understand the medical interpretation challenges for pain-related visits.

In summary, existing studies have provided insights into various factors such as age, gender, and language proficiency to account for interpretation inaccuracies. However, how these factors manifest in interpreter-mediated medical interactions are still unclear (Anazawa et al., 2012). More interactional studies are needed to determine the interaction-internal and language-specific factors that contribute to ineffective communication during medical interpretations, particularly for pain-related visits. Adopting conversation analysis (CA) methods, which allows microanalysis of medical interactions, this study aims to explore interactional features that account for interpretation inaccuracies in medical consultations involving Hmong patients. Such nuanced knowledge has significant implications for training medical interpreters.

Emerging CA research on pain communication has offer insights into patients’ practices discuss pain experiences (Wu, 2020), and clinician’ verbal and non-verbal practices for setting agenda for pain discussion (Hood-Medland et al., 2021), soliciting, exploring, and validating patients’ concerns (Cowell et al., 2021). Problems with using pain scales have also been analyzed (Jenkins et al., 2022), highlighting that the subjective nature of pain pose challenges for general patients, and these difficulties are compounded for those with language barriers. However, as far as we can ascertain, there is limited CA research on pain communication involving LEP patients, which is a research gap that our study addresses. Recognizing that inaccuracies in medical interpretation can differ depending on the language, we selected the Hmong language for this study. Previous research has shown substantial issues in medical interpretations for the Hmong population (Lor et al., 2021, Lor and Chewning, 2016). Therefore, Hmong serves as an ideal case for examining sources of interpretation inaccuracies in medical consultations involving Hmong patients telling pain stories, specifically interaction-internal and language-specific factors that challenge effective interpretation.

### 1.2. Hmong population and pain story communication

The Hmong population in the U.S. is approximately 368,000, with the largest population mainly residing in California, Minnesota, and Wisconsin (Pew Research, n.d.). The Hmong, a traditionally oral society with a recently developed a written language established for them by a Christian missionary in 1952 (Duffy, 2000). Yet, not all Hmong can read and write in Hmong, posing challenges for them to communicate their pain, specifically when they are required communicate their pain using numeric, faces, or visual analogs (Lor et al., 2024a). Coupled with their cultural differences in views of health and illness from Western biomedical concepts, as elucidated in the Spirit Catches You and You Fall Down, the Hmong experienced barriers to optimal pain care (Fadiman, 1998).

Lor and colleagues reported that Hmong patients communicate their pain using storytelling (Lor et al., 2020). They also found that clinicians have difficulty understanding Hmong patients’ stories about pain (Lor et al., 2020) and interpreters struggled to interpret the pain information for clinicians (Lor et al., 2022). However, no research has studied how Hmong patients’ stories about pain contribute to misunderstanding, where miscommunication errors occur, and how their pain expressions are overlooked during clinical encounters.

### 1.3. Analytical framework

Our study adopts a CA-informed approach. CA emerged out of ethnomethodology and has become a robust research method adopted in sociology, linguistics, and social psychology for the past five decades (Heritage, 2008). CA research is typical in their analysis of interactional mechanisms such as *sequence analysis, turn-turning, and repair* to uncover the workings of the social world (Hutchby and Wooffitt, 2008). As a research paradigm, CA is distinctive in its emic perspective and data-driven principle (Have, 2007). Briefly speaking, CA’s emic approach specifies that analytical claims need be based on what participants demonstrate within the interaction, instead of analysts’ own interpretation. Furthermore, CA’s data-driven principle requires analysts to start with what can be observed in the data instead of basing on theories or hypotheses.

Our study is framed within CA-informed research rather than adopting CA as the research paradigm (Have, 2007). We draw on CA insights on sequence analysis to identify pain communication sequences. We also draw CA literature on storytelling and turn-taking to analyze the discrepancies between the patient’s speech and the interpreter’s speech. However, when identifying interpretation errors, we do not adopt CA’s emic perspective, which views a communication problem as an error only if participants initiate and resolve a repair within the interaction itself (Hutchby and Wooffitt, 2008). Instead, our identification of interpretation errors is informed by existing empirical research and our cultural and linguistic knowledge (three out of four authors of this paper speak Hmong as a heritage language). In the context of interpreter-mediated interaction, participants are less likely to be aware of errors made by the interpreters. Therefore, it is necessary to rely on analysts’ cultural and linguistic knowledge in identifying such errors. Moreover, CA authors acknowledges the role of analysts’ cultural knowledge in developing analyses, especially when analysts are members of the same cultural group as the participants (Hutchby and Wooffitt, 2008).

Beside the CA literature on storytelling, we adopt Labov & Waletsky’s idea that a minimal story needs to have at least two actions that are temporally ordered (Labov and Waletzky, 1967), because we find the definition useful for us to identify pain stories at the initial stage. However, we do not adopt Labov’s narrative analysis approach. Instead, our analysis aligns with CA in its focus on turn-taking and multi-modality (Ta, 2021, 2022, 2023). Regarding turn-taking, a storytelling may take place in an extended turn of talk, which consists of multiple turn construction units (Ta and Filipi, 2020). A turn construction unit (TCU) is defined as the smallest unit of talk where a meaningful social action is accomplished (Hutchby and Wooffitt, 2008). It can be made of one word, one phrase, or one sentence. For example, “really” produced alone may be interpreted as an action of confirmation, doubt, or surprise depending on the intonation. This example not only illustrates how small a turn construction unit can be, but also highlights the use of intonation in producing and interpreting social actions. While any kinds of speech activities involve multimodal channels of communication, such as intonation, pitch, voice intensity, loudness, elongation, eye contact, and body movements, multimodal features are particularly important in storytelling (Stivers, 2008). They are not only essential for conveying the tellers’ emotions (affective stance) but also serve to provide hints regarding how listeners should make sense of and react to the story under telling.

### 1.4. CA research on interpreter-mediated medical interaction

From a CA perspective, interpreting is viewed as a multifaceted activity that goes beyond simply translating speech into a different language (Bolden, 2000; Raymond, 2014). Interpreters’ actions are influenced not only by what clinicians or patients say, but also by their understanding of the goals of the medical encounter (Bolden, 2000; Raymond, 2014), and by the dynamics of the turning-taking system involving multiple participants (Li, 2013).

CA research has also highlighted the role of interpreters as epistemic brokers who actively redesign content to align with the recipient’s understanding while ensuring the maintenance of mutual understanding between patients and clinicians (Bolden, 2018; Raymond, 2014). Specifically, Xu and Bridges (2022) showed that, to manage mutual understanding, interpreters engaged in reformulating statements, summarizing key points, and eliciting relevant expressions to optimize mutual understanding. Additional, research has also illustrated the use of multimodal resources such as gestures, body posture, and gaze in faciliating mutual understanding (Pasquandrea, 2011; Vranjes et al., 2019).

Beyond mediation of medical contents, interpreters also play a critical role in empathy communication. Farini (2015) found that interpreters’ actions prevented patients’ emotional expressions from being relevant in the interaction, thereby impeding emotion-sensitive care. Additionally, interpreters were found to regularly fail to render patient-initiated empathic opportunities, which referred to the patients’ talks that were relevant for clinician to express empathy (Krystallidou et al., 2020). These empathic opportunities either shifted in meaning or intensity during interpretation, potentially affecting a clinician’s ability to respond empathically (Krystallidou et al., 2020).

The present paper expands CA research in interpreter-mediated interaction by examining how problems with mutual understanding may arise in the context of pain stories involving Hmong patients, providing evidence on how interpretation problems may potentially impact empathy communication and other outcomes of the medical encounters.

### 1.5. Interpreter role and conceptualization of interpretation inaccuracy

According to the National Standards of Practice for Interpreters in Healthcare in the U.S., a professional interpreter renders all messages without adding, omitting, or substituting to ensure accurate and effective communication (National Council on Interpreting in Health Care, 2005). This perspective highlights the importance of both accuracy and effectiveness. While accuracy emphasizes the role of interpreter as a conduit, effectiveness implicates others important roles such as clarifier, cultural brokers, advocates (Hsieh, 2024). As *clarifiers,* they modify the patient’s language to ensure comprehension and explain terms that lack direct linguistic equivalents. In the role of a *cultural broker,* interpreters provide essential cultural context to enhance the accurate interpretation of messages. Meanwhile, as *advocates,* they go beyond interpreted interaction to actively support the patient, working to improve the quality of communication and health outcomes.

The multiple roles of interpreters can be understood in the light of three foundational concepts in translational studies, that is *equivalence*, *adequacy,* and *effect*. Hence, this study defines inaccurate interpretation in terms of issues related to equivalence, adequacy, and effect. *Equivalence* refers to the correspondence between the source text and the target text. It can be quantitative (focused on the amount of correspondence) or qualitative (focused on the nature of correspondence) (Kobenko and Ptashkin, 2014). Equivalence links to *semantic fidelity*, which refers to whether the interpreted message retains its semantic value in the target language (Rȩdzioch-Korkuz, 2023). A *semantic inaccuracy* (such as omitting or incorrectly translating a key medical term) disrupts the equivalence between the source and target message, leading to significant communication breakdowns. However, strict *semantic equivalence* can lead to errors in communication, for example, directly translating idiomatic phrases can result in misunderstandings between clinicians and patients.

To address the limitation of *semantic equivalence*, scholars have proposed other forms of equivalence such as functional, pragmatic, and cultural equivalence. *Functional equivalence* refers to the concept of achieving the same function or effect in the target language as in the source language, rather than focusing solely on literal equivalence (Zhou, 2022). Achieving *pragmatic equivalence* means that the translation should evoke the same response and understanding in the target audience as the original text does in its audience (Baumgarten, 2022). *Cultural equivalence,* in contrast, refers to the process of finding a target language equivalent that accurately conveys the cultural context and meaning of the source language (Hamamoto, 2023).

Two others concepts related to *interpretation inaccuracy* are *adequacy* and *effect* in translation and interpretation. *Adequacy* concerns whether the interpretation serves its communicative function and meets the needs of the participants in the interaction (Sosnin et al., 2022). *Inadequacy* links to *functional inaccuracy* (Rȩdzioch-Korkuz, 2023), which arises when the interpreted message fails to serve its intended purpose—whether that is informing or reassuring the patient. This can occur when the interpreter presents a message in a manner that fails to align with the speaker’s communicative goals.

*Effect* refers to the impact that the interpreted message has on the interaction, particularly in terms of how it influences the audience (Huijuan, 2007). *Effect* links to *pragmatic equivalence* and *cultural equivalence* (Baumgarten, 2022). Failure to find pragmatic equivalents can lead to *pragmatic inaccuracy*. For example, an interpreter’s failure to accurately convey the tone of the message results in failure to preserve the speaker’s intended emotional impact, which can alter the outcome of a medical interaction. Similarly, failure to interprete culture-loaded words can lead to *cultural inaccuracy* which can influence the overall effect of an interpreted message (Hamamoto, 2023).

Building on the translational studies reviewed above, this study defines inaccurate interpretation in terms of issues related to equivalence, adequacy, and effect.

## 2. Research design and methods

This study was approved by our university’s institutional review board. This study uses the audio recorded data from a pilot intervention study (see Lor et al. (2024a) for details of the study including information on the clinical setting and the characteristics of the sites, clinicians, patients, and interpreters).

### 2.1. Design

In the pain communication study (Lor et al., 2024a), a static group comparison design was used to collect data from 20 patient, interpreter, and clinician triads under usual care (i.e., interpreter using verbal pain descriptions), followed by another 20 triads under the intervention (i.e., interpreter using verbal pain descriptions and the Pain Assessment Information Visualization [InfoViz] tool). The InfoViz Tool comprised six culturally appropriate faces pain severity scale, a body diagram, and 11 pain quality icons to represent each pain quality metaphor which were co-created with Hmong with LEP and interpreters (refer to Lor et al. (2024a) for more details of intervention). For this study, we only included a total of 27 visits for our analysis as they focused on pain discussions. Having video recordings of these visits would be ideal for capturing body language; however, this was not possible because we did not anticipate their importance during initial stages of the project, and therefore did not obtain participants’ consent for video-recording.

### 2.2. Sample

Participants included in this study were LEP Hmong patients, interpreters, and clinicians. The inclusion criteria for LEP Hmong patients involve identifying Hmong individuals aged 18 and above, who have indicated Hmong as their primary language preference, and self-report pain. Interpreters were included if they indicated interpreting for the Hmong language and were willing to participate in the study. Clinicians could include medical doctors, osteopathic doctors, nurse practitioners, physician assistants, or residents. Clinicians were included if they indicated having at least one or more LEP Hmong patients.

### 2.3. Data collection

For both phases during the clinic visit, the conversations between a Hmong patient, medical interpreter, and clinician were audio-recorded. Although for the intervention phase, the tool was used, clinicians were not provided with specific instructions on how to use it because we wanted to understand the natural approach and action. See Autor for details of the study (Lor, Li, et al., 2024). The audio recordings of patient, interpreter, and clinician interactions during the visit were used for this study.

### 2.4. Data transcription and analysis

The audio recordings were first transcribed verbatim by the second and fourth authors. First, 93 storytelling extracts were identified in our data set. Next, these extracts were refined by the second and first authors using the Jeffersonian system (Jefferson, 2004). This transcription system can capture vocal features while allowing the readability of the words. Like many other transcription systems, the Jeffersonian system is not perfect; it cannot capture all of what actually happened (e.g. the accuracy of the elongated sounds, pitch, and voice intensity). However, at a practical level, it can capture sufficient details that are helpful for facilitating analysis and discussion (Walker, 2012).

In the transcripts, each turn construction unit (TCU) occupies a separate line. This treatment facilitates the intelligibility of the multi-unit turns. Unlike sentences in written language, turn construction units are not predetermined by syntactical rules, but are locally determined through the deployment of lexical, syntactical, and prosodic resources (Hutchby and Wooffitt, 2008). Therefore, the identification of turn construction units requires careful consideration of the local context. It is important to note that commas, full stops, question marks, and other syntactical symbols are not used in a conventional sense. See Table 1 for the meanings of symbols essential for the analysis in this study. For other symbols, please refer to Jefferson (2004).

Initial data analysis was conducted in monthly team meetings over the course of seven months. An inductive procedure of analysis was adopted: the team first examined closely randomly selected transcripts of pain communications and made notes of interesting features, e.g. the use of storytelling, vocal features by the patients, and features of the interpretations by the interpreters. For example, we examine individual instances where the meaning conveyed by vocal features is not accurately reflected in the interpretation, whether lexically or otherwise.

## 3. Results

### 3.1. Overview

The analysis sample included 93 storytelling episodes produced by 27 patients. Note that each storytelling episode can start either with the clinician’s turn that elicits the patient’s storytelling, or with the patient’s turn in which the patient initiates the storytelling without being elicited, and end with the clinician’s response to the storytelling. The patient’s storytelling turn can minimally consist of two TCUs, which describe two temporally ordered actions. For example, a story may be as short as “*my back hurt at night, I had to get up and give it a massage”*.

All patients engaged in telling pain stories with a median of 3 stories per patient (min = 1, max = 8 stories). Overall, Hmong patients use emphatic voice (intensity, loudness or high pitch), elongation, and repetition to effectively convey the pain severity, the level of urgency or importance of the pain concern, and associated emotions within their pain stories. Patients’ descriptions of pain frequently involve three major features, such as empathic voice, elongation of sounds, and repetition of descriptor words, which refer to words that describe pain. While all patients used emphatic voice in descriptor words, with the highest total counts of 1131, there were variations in its frequency of use (a median = 39 [min = 4, max = 125]). Elongation was identified in 81 % of the patients (22/27), with a total number of 117 counts. Repetition was identified in 89 % of the patients (24/27) with total 143 counts. Features of patients’ pain stories are summarized in Table 2. Examples of these features will be analyzed in 3.3.

We find that the pain severity and associated emotions, as expressed by the vocal features as listed above, are often not accurately conveyed in the interpreted stories. Table 3 shows that patients’ number of pain description words produced with intensity, loudness, and high pitch is 7.5 times higher than that of what interpreters actually interpreted to clinicians (1131/150). Note that we did not include words that were not used to describe pains in the patients’ speeches and interpreters’ speeches. The rate for elongated words is 2.9 times (117/40) higher than what interpreters conveyed.

Moreover, interpreters frequently summarized patients’ stories instead of interpreting all the messages conveyed by the patients. Of all 93 storytelling episodes, interpreters produced only 353 TCUs, which is half of the number produced by patients (n = 729). We do not suggest that interpreters must translate word for word to ensure accuracy. From a CA perspective, TCU is the smallest unit of meaning where social action occurs; therefore, it is essential to convey the meaning of the patient’s social action in each TCU. CA’s notion of social action aligns with the concept of function in Speech Act Theory, emphasizing that language serves social purposes beyond a mere representation of knowledge (Searle, 1979).

The relationship between TCUs and functions is not always one-to-one; a single TCU can serve multiple functions. For instance, *“my back hurts"* may both elicit empathy and seek medical advice, depending on how and when it is said in the sequence of talk, but the interpreter may only convey one function. Likewise, an interpreter can condense multiple functions in one TCU, thus in individual cases, condensing interpretations into fewer TCUs may not be problematic. However, the frequency of condensing patients’ stories on a large scale as shown in Table 3 may risk obscuring important aspects of patients’ pain experiences, as illustrated in our qualitative analysis of conversational extracts. Our findings reveal that such practice can lead to inaccuracies, limiting opportunities for empathy and addressing patients’ concerns.

### 3.2. Analysis of interpretation inaccuracies and their potential consequences

#### 3.2.1. Inaccurate interpretations impeding empathic communication

Extract 1 is a typical example that demonstrates how interpreters summarize the patients’ pain story and fail to accurately convey the pain severity as expressed through patients’ vocal features, which in turn impede empathic communication.

In response to the clinician’s question about the shoulder (lines 1–2), the patient launches a storytelling, detailing the pain experiences, the bodily locations of pain, and the times when the pain occurs between lines 8 and 27. However, the interpreter does not translate the patient’s story closely line by line. The patient tells the story in ten TCUs while there are only four TCU in the interpreter’s version). As a result, some contextual details of the pain experience, i.e. the time context (lines 10–13) and bodily locations (lines 15–27), do not come through in the interpretation to the clinician (lines 28–32).

Moreover, various aspects of the pain experience expressed through the patient’s vocal features are not interpreted accurately (see the notations for these features). For example, the elongation, loudness and intensity of the words NTEV: (=long) (line 10), and NYAV (=heavy) (line 12), NQA (=lift/carry) (line 23), and NCEV (=reach) (line 22) express the patient’s affective stance, and work to intensify the circumstance where the pain occurred, but the meaning of these features is not conveyed by the interpreter (lines 28–32). Worse of all, the intensity of pain expressed the word “mob” (=pain or hurt), repeated throughout the patient’s story, produced in loud voice (lines 15 and 19), and with high pitch and intense and elongated voice in line 17, also do not come across in the interpretation (lines 28 and 32). Here, the interpreter simply relays the information in a matter-of-fact manner: it hurts when I have to do heavy lifting (line 30).

When patients display pain or emotional distress, it provides opportunities for clinicians to empathize and build therapeutic with patients. However, here in line 33, the clinician does not offer any comments to empathize with the patient, instead, the clinician merely clarifies factual information about the time of the pain occurrence. Across our data corpus, no case of empathy display is found in clinicians’ responses to patients’ pain stories.

In this extract, the patient’s pain story may serve dual functions: seeking both empathy and medical advice. However, the clinician focused solely on the medically relevant information while failing to acknowledge the emotional aspect. This lack of empathy cannot be attributed solely to poor interpretation; other factors, such as the dynamics of triadic interaction, the clinician’s priorities within the constraints of time and space, may also influence their response.

It is also important to note that a lack of displayed empathy from the clinician does not indicate a serious misunderstanding issue. In fact, the clinician’s response—seeking confirmation about the timing of the pain experience—demonstrates that they correctly understood certain details of the patient’s condition. This suggests that the interpreter was successful, at least partially, in facilitating the clinician’s comprehension.

**Table:** 

Extract 1: 1015E.1.	
1	C: what, (0.3) can you (0.2) tell me a little bit more about (0.3)
2	what’s going on with your shoulders?
3	I: awm > koj sim piav qhov me ntsis txog koj ob sab xub pwg rau kuv<?
4	**yes can you tell me a little about your shoulders?**
5	P: kuv ob lub xub pwg no ma,
6	kuv > KUJ tsis paub < hais tias ua cas thiab,
7	**I do not know why that is either,**
8	ua hauj lwm ces nws-
9	**I’ve been working then it-**
10	ua huaj lwm NTEV: zus zuj mus ces zo li pib mob zus zuj xwb na yom?
11	**the longer I’ve been working it seems to just start gradually hurting more?**
12	ces tas sim no ces zoo li ah koj ua tej qhov huaj lwm NYAV no ces,
13	**so right now it seems like when I do heavier work,**
14	(0.2)
15	MOB qhov koj qoj mus los (.) qoj nraum no na,
16	**it hurts at the hinge joint where it moves back and forth out here,**
17	no ces: ^mob::: hauv ua koj mloog mas zoo li,
18	**then it hurts on the inside where I feel like,**
19	>MOB hauv qhov ua tus pob txha sib qoj,<
20	**it hurts inside where the joints are moving,**
21	= tab sis kuj tsis paub tias puas yog qhov hauv los qhov twg thiab,
22	**but I don’t know if it’s the inside or where else either,**
23	ces koj NQA li no ces koj (.) NCEV tsis tau tes mus sab nraum li,
25	**so if I lift like this, then I am not able to reach my hands behind my back like this,**
26	ces > kawg li no xwb < no na.
27	**so it just stops here.**
28	I: >awm, yeah so< ^I don’t know um how lo:ng maybe it’s-
29	it’s hurting because of overuse from working,
30	but now it- it- it hurts when I have to do like heavy lifting
31	or I- I can’t reach backward um with the range of motion it feels like
32	(0.2) um there’s pulling in, pulling in the ^joints (0.2) of the shoulders
33	C: u^mm: (0.5) this was like (0.3) a: a ^year ago?
	(Note: P = patient, C = clinical, I = Interpreter)

#### 3.2.2. Inaccurate interpretations leading to concerns unaddressed

In the following two extracts, besides misrepresenting the severity of the pain, the interpreter failed to articulate the patient’s additional concern, which resulted in the concern being unaddressed by the clinician.

Between lines 1–6, the patient tells a story with three TCUs: 1) she’s unable to fall asleep, 2) she’s in severe pain, 3) she has to wake up to massage herself. Note that this is the second time the patient complains about her sleeping. However, in line 7, the interpreter summarizes the story in one TCU, and do not mention the sleeping problem. The patient puts emphasis on “mob:” (=hurt) through elongated and intense voice (line 3), and on “ZUAJ” (=massage) through loudness (line 5), but again the meaning of these features is not conveyed the interpretation. After this, the patient continues to provide details about the frequency of having to massage herself (lines 8–9), the effect of massage on the pain (lines 10–13), and reinforces the message that she always has to do that (lines 14–15). Here, the frequency of massaging is depicted by repeating the word “zuaj” (=massage) four times (line 8) and also through the conclusion at the end of the turn (lines 14–15). However, the interpreter does not convey this information at all. Instead, they only focus on the positive outcome of the massage on the patient’s sleep (line 16). As a result, the message about the unpleasant experience of sleep disruptions is overlooked, which accounts for why the clinician does not address the sleep issue in the response in lines 17–20. Instead, the clinician further explores the patient’s pain experience, using the pain assessment visualization tool. Notably, the misinterpretation of the patient’s concern may be partly due to the way the concern is originally articulated. Throughout her recount of pain experience in extract 2, the patient uses “koi” (=you) to refer to herself, which may make her speech less personal and assertive.

**Table:** 

Extract 2–1036E.300.	
1	P: TSAUG tsis taus zog ces,
2	**unable to fall asleep now,**
3	koj mob: DHAU lawm ces,
4	**you’re hurting too much,**
5	koj sawv los ZUAJ,
6	**you would wake up to massage,**
7	I: Because it’s so painful that she has to get up and kind of massage those area.
8	P: Awm koj > zuaj zuaj < es,
9	**yes you constantly massage it a lot,**
10	zoo nkaus li IB: nyuag zoo zog,
11	**then it seems to feel a little bit better,**
12	ces koj pw ces (.) koj thiaj pw taus,
13	**then when you sleep, you’re able to sleep better,**
14	na (.) ua li ntawd nkaus xwb as.
15	**so just always have to do that.**
16	I: and then she will feel a little bit better before she falls asleep and-
17	C: um if I ask you-
18	I see the picture,
19	if I ask you does it hurts in the muscle or the joint pain the bone.
20	would you be able to tell me which one?

About 1 min after the above extract, the sleep concern comes up again for the third time. In extract 3, the patient retells her pain experience again: the pain hurts her so much because she has a very hard bed (lines 73–76); and when she is in pain, she is not able to fall asleep (lines 77–78); she has to wake up and massage herself (lines 79–80); and there are places she is unable to massage (lines 81–82). The patient continues to detail the movements that hurt her arm muscles (lines 84–85), and the movements that help the pain (86–87), and states that the massage helps her sleep (lines 90–91).

Many details that the patient provides in the storytelling are not captured in the interpretation in lines 83, and 94–95. Here, the interpretation only focuses on the main event - the patient massaged, and the massaging helped and the interpreter does not include the details that convey the nuisance of having to wake up and do massage constantly. As a result of this misrepresentation, the clinician’s response only focuses on the positive outcome of the massage and does not discuss the sleeping concern (line 96).

**Table:** 

Extract 3–1036E.392.	
73	P: ces tej thaum mob heev DHAU as,
74	**so sometime when it hurts too much,**
75	ces kuv muaj ib lub txaj nws > tawv tawv < na yom?
76	**so I have a bed that’s very hard,**
77	ces kuv pw tsis taus ces,
78	**when I’m unable to sleep,**
79	kuv sawv los ZUAJ xwb os.
80	**I just wake up to massage.**
81	sab no kuv zuaj tsis lais (.) ces kuv yeej ua no xwb os.
82	**this side here I’m unable to massage it so I just do it like this.**
83	I: [she said that sometime when it’s very painful]
84	P: [kuv ua no ces kuv cov leeg MOB: li no],
85	**I massage it like this then my muscles hurt like this**
86	ces ua no mas NRUJ nrees,
87	**so if I do it like this then it’s very tight,**
88	ces mam taug.
89	**then the pain will improve.**
90	= ces kuv thiaj mas pw TAUS,
91	**then I will be able to sleep,**
92	NO xwb os::.
93	**just like that.**
94	I: she’ll wake up and then kind of self-massage or pressed against
95	that hard space to kind of loosen it up to allow her to go to sleep and-
96	C: oh does it– it sounds like the massage is helpful

## 4. Discussion and conclusion

### 4.1. Discussion

We found discrepancies between Hmong patients’ original pain stories and their interpretations by medical interpreters which are associated with two common aspects in Hmong patients’ pain communication 1) the frequent use of storytelling to describe pains and 2) the reliance on vocal features such as elongation, intensity, loudness, pitch, and repetition to express pain severity. Our findings confirm that Hmong patients commonly use storytelling to convey their pain experience (Murphy et al., 2023), and expanded on their concerns about medical interpreters’ inadequate language proficiency (Lor and Chewning, 2016; Lor et al., 2024a).

Hmong patients recounted their pain experiences in extended storytelling with multiple-turn construction unit. However, interpreters usually summarized the original stories using fewer numbers of TCUs. At single case level, doing interpretation into fewer TCUs is not necessarily problematic as interpreters can potentially convey the patients’ messages in a condensing way. However, at a large scale when the reduction practice is repeatedly used, it may potentially occlude important information about patients’ pain experiences as shown in analysis of the conversational extracts. As a result of poor interpretation, the clinician in extract 1 received inaccurate information on the time context, the precise location of pain, and severity of the pain. Similarly, the clinician in extract 2–3, did not receive information about the frequency of pain-associated sleeping problem, and the intensity of pain. This finding echoes with other studies which found that interpreters usually provide summary interpretations, selecting details that are medically relevant and omitting emotional information (Bolden, 2000; Hofer, 2020; Kai, 2013; Theys et al., 2020).

Several factors may have contributed to the interpreters minimizing patients’ pain narratives including time constraints during clinic visits (Kai, 2013), interpreters’ understanding of what is considered relevant to the ongoing task (Bolden, 2000), and perception that clinicians prefer concise answers over detailed stories, and potential lack of experience (Flores et al., 2012).

This study also highlights Hmong patients’ reliance on vocal features—tone, pitch, and intensity—in expressing pain, underscoring the need for culturally sensitive interpretation of pain communication. This reliance may be due to the low variety of degrees of adverbs to describe pain severity, quality, or characteristics in the Hmong language. For example, it does not have equivalents of English adverbs for describing the seriousness of pain such as “severely," “extremely," or “persistently.” Hence, Hmong patients convey such pain information through vocal features since the Hmong language is a tonal language, the use of vocal features conveys key meanings. Therefore, Hmong-speaking interpreters may not have understood the linguistic meanings of these vocal features, leading to decreased or misinterpreted communication of these features to clinicians. Furthermore, in a culture like the Hmong where people do not explicitly express their needs with statements like “I need" or “I want," they are more likely to not receive the necessary care. This is evident in cases where, if concerns are not clearly articulated—such as saying “I have trouble sleeping because of pain"—the patient’s sleep issues may go unaddressed. When compounded with challenges in interpretation where such nuances are not effectively interpreted, it becomes even harder to draw the clinician’s attention to the patient’s needs.

Analysis of the clinicians’ responses shows that misinterpretation of pain stories may lead to missing opportunities for displaying empathy. Empathy has been highly emphasized in primary care (Derksen et al., 2013). Empirical evidence shows that clinicians in encounters without interpreters regularly empathize with patients either verbally or via multimodal channels when patients display pains or recounting their emotional experiences (Davidsen and Fosgerau, 2014; Ruusuvuori, 2005; Weatherall and Keevallik, 2016). CA research has shown that when patients express pains and emotions, clinicians usually display empathy by claiming understanding of the patients’ experiences, naming the emotions (Tietbohl, 2022), or sharing similar experiences (Versteeg and te Molder, 2016). These types of responses are not found in the data set of this study.,. It is important to recognize that a lack of empathy display is not solely a result of poor interpretation. Other factors, such as the complexities of triadic communication, the clinician’s priorities, and the time and space constraints may also shape their response.

Inaccurate interpretations may also result in missing opportunities for addressing patients’ concerns. Several literature reviews have reported that inaccurate interpretations can lead to incomplete assessments and compromised treatment planning and referrals to specialty care (Bauer and Alegría, 2010; van Rosse et al., 2016).

Overall, our study offers new insights into the interpretation issues in interpreter-mediated pain communication by bringing lenses from CA and translational studies. First, through the CA lenses, we located two potential problematic practices, the reduction of using fewer TCU in storytelling, and the missing interpretation of vocal features as explicated. Second, through the concepts of equivalence, adequate and effect from translational theories, we demonstrated the complexity of interpretation accuracy. While semantic accuracy was usually achieved (except for the inaccurate equivalent to pain location in extract 1), it is more challenging to attain functional accuracy and cultural equivalence. Certain functions of the patients’ storytelling were successfully conveyed (e.g., informing clinicians about the pain), the function of eliciting empathy did not come through, and the cultural-linguistic practices of on vocal features to describe pain pose challenges for interpretation.

### 4.2. Limitations

Due to data limitations, this study does not examine facial expression, gestures, and sitting positions of the participants, which are undoubtedly important in pain communication. Using video-recorded data would enable discoveries of other sources of misinterpretation. For example, inaccuracies may happen due to failure to recognize certain body language features, or due to lack of eye contact. Future research can draw on video-recordings to uncover how interpreters attend to and convey embodied actions, and how they can use multimodal resources in facilitating mutual understanding between patients and clinicians in pain communication. Another limitation of this study is the lack of comparative data for conversations unrelated to pain or for clinician-Hmong patient interaction without interpreters, which could provide additional insights into communication dynamics in different contexts. Future studies could explore and compare the differences in features such as empathetic voice, elongation, and repetition in conversations related to pain versus those unrelated to pain. Likewise, comparing pain communications with and without interpreters could yield valuable insights.

### 4.3. Conclusion

The use of story-telling and vocal resources plays important roles in describing the pain experiences and expressing pain severity among Hmong patients. These features are particularly challenging for Hmong interpreters due to the reliance on vocal resources in conveying meaning in the Hmong language.

### 4.4. Practice implications

There are several practical implications that we could draw from this study’s findings. First, interpreter training could emphasize that interactional practices are multi-functional and context-dependent; therefore, it is important to closely attend to contexts, and skillfully convey multiple layers of meaning. Second, the training could include the significance of recognizing culturally distinctive pain expressions when working with culturally diverse populations. Since interpreters act as crucial mediators, their ability to convey multimodal pain expressions accurately is essential for effective communication.

However, our study does not suggest that interpreters need to closely translate utterance by utterance like a machine or use the same vocal features the same as the patients, which is not only unrealistic but also undermines the multifaceted role of medical interpreters. But rather our study supports expanding the role of the interpreters to also act as an agent of empathy, assisting clinicians to understand patients’ experiences, and convey empathy should be considered (Gutierrez et al., 2019). These recommendations both highlight the importance accurate interpretation, and recognize the active role of interpreters in assisting patients and clinicians to achieve their communicative goals and enhance quality of care (Hsieh, 2024).

## Figures and Tables

**Table 1:** Transcription codes and meanings of symbols.

Transcription Codes	Transcription Definition
.	Falling intonation at the end of TCUs
?	Rising intonation at the end of TCUs
,	Slightly rising intonation at the end of TCUs
Underlining	Stress or intense voice
UPPERCASE LETTERS	Talk with loudness
^	Upward in pitch, e.g. ^ carrot
:	Elongation or stretching of the sound, e.g. pa:ssing
**Bold text**	Researchers’ translation of patients’ talk

**Table 2:** Patients’ use of vocal features in pain stories.

Features	Number of patients producing the feature	Counts per patient			Total counts for all patients
		Median	Min	Max	
Emphatic voice (Intensity, high pitch, or loudness)	27/27	39	4	125	1131
Elongation	22/27	2	0	28	117
Repetition	24/27	4	0	14	143

**Table 3:** Comparison of patients’ story features to the interpreters’ actual interpretation.

Features	Total Number of Features Within Storytelling by Patients	Total Number of Interpreted Features Within Storytelling by Interpreters
Empathic voice (Intensity, high pitch, and loudness)	1131	150
Elongation	117	40
Repetition	143	0
Turn construction unit	729	353

## Data Availability

The authors do not have permission to share data.
